# Unintended evolutionary consequences of a minimum landing size regulations: Evidence and implications of fisheries-induced evolution

**DOI:** 10.1371/journal.pone.0343706

**Published:** 2026-03-02

**Authors:** Airam Guerra-Marrero, Javier Baixauli-Rodríguez, Ana Espino-Ruano, David Jiménez-Alvarado, Lorena Couce-Montero, Ángelo Santana-Del Pino, José J. Castro

**Affiliations:** 1 Faculty of Education, Universidad del Atlántico Medio, Las Palmas de Gran Canaria, Spain; 2 IU-ECOAQUA, Universidad de Las Palmas de Gran Canaria, Edf. Ciencias Básicas, Campus de Tafira, Las Palmas de Gran Canaria, Las Palmas, Spain; 3 Departamento de Matemáticas, Universidad de Las Palmas de Gran Canaria, Campus Universitario de Tafira, Las Palmas de Gran Canaria, Las Palmas, Spain; Central Marine Fisheries Research Institute, INDIA

## Abstract

A total of 725 historical photographs taken between 1940–2022 were analysed to assess the temporal variations in target species and the size-frequency distribution of recreational fishing catches in the Canary Islands. A significant shift from predominantly single-species fishing to a more diverse, multispecies approach was observed, particularly since 2000. Species such as *Dentex gibbosus*, *Diplodus cadenati*, *Epinephelus marginatus*, *Lithognathus mormyrus*, and *Sparisoma cretense* showed consistent size reductions over the decades. The analysis revealed discrepancies between current Minimum Landing Sizes (MLS) in the Canary Islands and the lengths at which these species reach sexual maturity (L50), or the length at which 50% of fish are mature. Except for *Diplodus cadenati*, the MLS for the examined species are smaller than their respective L50 values, indicating that individuals are often harvested before reproducing. The use of the L50 as a standard for reference length can trigger a fisheries-induced evolution. This occurs because the extractive activities resulting from this standard promote reduced fish size and earlier maturation, which could lead to lasting evolutionary changes in fish communities. Our study underscores the need to reevaluate minimum landing size (MLS) regulations and seek a transition towards L95, to ensure they are aligned with the reproductive biology of target species, thereby promoting sustainable fishing practices and the conservation of marine biodiversity in the Canary Islands. Even so, this proposed improvement and the results of this study should be combined with a Harvest-slot length limits (based on species-specific ecological and demographic studies) to ensure the survival of megaspawners.

## 1. Introduction

Overfishing represents one of the main threats to the sustainability of marine ecosystems and global food security [1,2]. The percentage of fish stocks exploited at biologically unsustainable levels increased from less than 10% in 1974 to 37.7% in 2021, highlighting a growing and alarming trend in the overexploitation of fisheries resources [3]. This phenomenon not only affects target species but also negatively impacts species such as sharks and rays, by altering food webs and compromising the ecological integrity of marine ecosystems [4].

In response to overfishing, fisheries management began to take formal shape in the 20th century with the goal of regulating the exploitation of marine populations [5,6]. A major milestone achieved in this discipline was the work of Beverton & Holt [7], who introduced models to estimate the Maximum Sustainable Yield (MSY) and provided theoretical justification for implementing Minimum Landing Sizes (MLS) as a measure to maximize yield per recruit.

Interestingly, the origins of MLS regulations go back even further. In 1558, under the reign of Queen Elizabeth I of England, regulations were enacted to prohibit the capture of Atlantic salmon (*Salmo salar*) under 16 inches, with the aim of protecting juvenile individuals in British rivers [8]. Since then, MLS has become a widely used fisheries management tool, designed, as Solomonic judgement, to ensure that at least 50% of the population (L50) reaches sexual maturity thus potentially contributing to recruitment before being vulnerable to harvest.

However, this strategy is not without consequences. The inherent selectivity of MLS—protecting smaller individuals while preferentially removing larger ones—can induce unwanted evolutionary responses in the life-history traits of exploited species. Over time, these artificial selection pressures may promote populations with higher growth rates and smaller reproductive sizes, altering the genetic structure of fish populations. Several studies have warned of the reduction in genetic variability caused by selective fishing, which diminishes the adaptive capacity of populations to cope with environmental changes, disease, and other pressures [9,10].

The loss of genetic diversity is further exacerbated by additional factors such as pollution, habitat degradation, and climate change, which interact with overfishing to further weaken the resilience of marine ecosystems [11–13]. This genetic diversity is essential for adaptive evolution and for the persistence of species in changing environments [14,15].

There is growing empirical evidence that fish currently caught are significantly smaller than those recorded in past decades [16,17]. Guerra-Sierra & Sanchez-Lizaso [24] describe this phenomenon as ‘genetic overfishing’ when fishing selectively eliminates larger individuals, so that over time undesirable genetic traits from a human point of view are selected, such as those which slow growth or cause reproduction to smaller sizes, which adversely affect fecundity. It is not known on what time scale this type of phenomenon can occur, but at least from a theoretical point of view it should be considered [18].

Small scale fishery in the Canary Islands is a data-poor system, and only from 2006 onwards did the systematic collection of catch records by species begin after the establishment of first sale points by the autonomous government of the islands. In 1986, MLS regulations were first introduced for more than twenty species in the Archipelago, but unfortunately the lengths of the individuals caught have never been recorded by the registration system, so it is not possible to determine whether fishing activity has produced changes in the length structures of the fished species. The present study uses data collected through citizen science —historical photographs of recreational fishing catches—to analyse: (i) shifts in target species over time, and (ii) variation in captured sizes. MLS regulations, originally designed as a conservation tool, may have contributed to demographic and evolutionary changes in some fish populations, that might be maladaptive in the contest of present and future ecological scenarios.

## 2. Materials and methods

Changes in the size structures of fish caught by the recreational fishing community along the coast of Gran Canaria Island (Canary Islands, Spain) over the last eight decades (1940–2022) were quantified using historical photographs. A total of 725 photographs were collected with the help of recreational fishing associations of all fishing modalities (shore fishing, spearfishing and boat fishing), many of which are displayed in their showcases as souvenirs of competitions, but also from fishing tackle shops and individual fishermen who allowed us to scan and copy their photos for later analysis in the laboratory.

The use of photographs for size estimation is a complex task. The total length (TL) of the fish was calculated using indirect measurement methods based on objects of known size, following the procedure described by Jiménez-Alvarado et al. [17]. Fish measurements in the photographs were carried out with the help of the ImageJ software [19]. All photographs that lacked a reference object or in which the object’s position did not allow accurate measurement were discarded.

The collected data—fish length and the number of species—were organized by year. A Generalized Additive Model (GAM) was used to establish relationships between size and year, as well as changes in the target species. The effective degrees of freedom (edf) obtained from the models were equal to 1 in all cases; therefore, linear regression were also fitted to the data, estimating species-specific mean change in size per year.

A significant trend in the size (total length) of *Dentex gibbosus* (Rafinesque, 1810; pink dentex), *Diplodus cadenati* de la Paz Bauchot & Daget, 1974 (Moroccan white seabream), *Epinephelus marginatus* (Lowe, 1834; dusky grouper), *Lithognathus mormyrus* (Linnaeus, 1758; striped seabream), and *Sparisoma cretense* (Linnaeus. 1758; parrotfish) were specifically analysed, due to that were the most frequently presented in the photographs. For the remaining species analysed, a reasonable number of photographs were not obtained to draw robust conclusions (sometimes only 2 or 3 photographs per species, see Annex 1). Although other species also appeared in the photographs, there were not enough data to conduct a robust temporal analysis for them (Annex 1). The change in catch composition—i.e., whether the number of species varied—was also studied over the years.

All analyses were performed in R statistical software (version 4.3.0) [20]. Generalized additive models (GAMs) were fitted using the “mgcv” package [21].

## 3. Results

**Fig 1:**
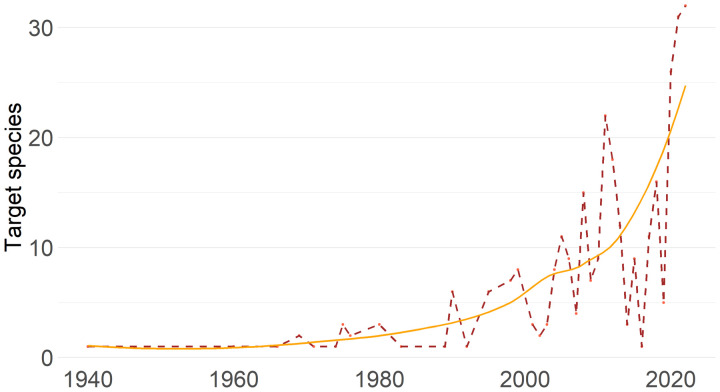
Temporal evolution of the number of target species over the years in photographs provided by recreational fishermen between 1940 and 2022. Orange line: trends; brown hatched line: real number of target species along the years.

The analysis of the photographs has revealed a notable increase in the number of target species caught by fishers over the years, shifting from a predominantly monospecific fishing practice to a much more diverse and multispecific fishery. Although it is true that in the early years of the analysed time series (1940s to 1980s) taking photos was more difficult and not all fishermen had cameras to immortalize their catches, and this may be introducing a significant bias into the data, particularly exaggerating a greater tendency to photograph large specimens, when analysing the data obtained only with photos taken from the year 2000 onwards, when photography was already more democratized by mobile phones, a decreasing trend in the average size of the specimens caught is observed (Fig 1), even with a steeper slope than in the period prior to that year. Moreover, after this year the number of target species has experienced substantial growth, rising from less 10 species to over 30. This reflects a significant shift in fishing strategies and objectives. Table 1 presents the five selected species.

**Table 1:** Number of samples (n), mean Total Length (TL, cm), and standard deviation (SD) for each species and time period analysed.

	1960-1969		1970-1979		1980-1989		1990-1999		2000-2009		2010-2019		2020-2022	
Species	n	TL (SD)	n	TL (SD)	n	TL(SD)	n	TL (SD)	n	TL (SD)	n	TL (SD)	n	TL (SD)
*Dentex gibbosus*			2	87.5 (3.5)	2	77.5 (10.6)	1	70.6			11	45.2 (7.9)	12	31.3 (5.8)
*Diplodus cadenati*			1	35.0			5	24.3 (3.6)	11	23.3 (6.8)	45	19.8 (4.6)	8	14.7 (2.9)
*Epinephelus marginatus*	13	95.2 (25.5)	19	94.5 (20.8)	3	59.8 (13.1)	2	48.5 (2.1)	3	33.8 (1.3)	11	52.3 (15.1)	4	19.5 (2.1)
*Lithognathus mormyrus*			1	31.3			6	24.1 (2.8)	4	26.9 (2.5)	2	17.0 (0.2)	4	17.7 (1.7)
*Sparisoma cretense*	1	49.0	3	43.8 (3.2)	4	39.8 (1.0)	4	36.4 (2.3)	20	33.1 (7.9)	61	26.2 (7.2)	7	18.6 (4.1)
*Total*	14	91.9 (27.4)	26	83.37 (28.2)	9	54.8 (17.6)	18	32.2 (13.0)	38	29.7 (8.1)	130	27.7 (12.5)	35	22.0 (8.0)

**Fig 2:**
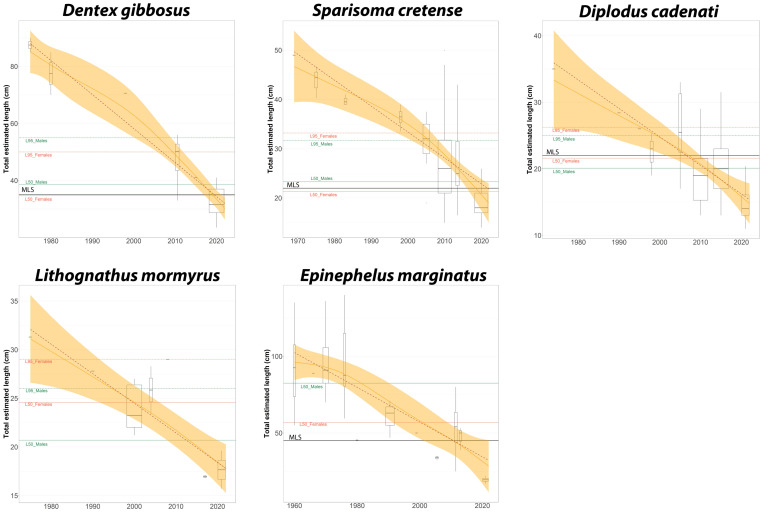
Variation in the average total estimated length of *Dentex gibbosus*, *Diplodus cadenati*, *Lithognathus mormyrus*, *Sparisoma cretense*, and *Epinephelus marginatus* photographed between 1960 and 2022. The brown dashed line represents the linear regression fit, while the orange line shows the non-linear GAM model fit (confidence band). Horizontal lines indicate size reference points: solid and dashed lines represent the L50 and L95, respectively, for females (orange) and males (green). L95 is not available for *Ephinephelus marginatus*, which is why it is not shown in the figure. The solid black line represents to the legal Minimum Landing Size (MLS) in the Canary Islands. *Lithognathus mormyrus* does not have a MLS regulation.

The GAM analysis of the five analysed species reveals that this decline has not occurred at a constant rate. Between 1940 and 1970, the reduction was slower, but from that point onward, total length began to decrease more sharply, reaching a low around the year 2000 (Fig 2). Since then, the rate of decline stabilized, and in some years, there was even a slight increase. However, after 2010, the downward trend resumed and has continued to the present day (Fig 2).

A pronounced decrease in size among years were found for four of the analysed species, the pink dentex *Dentex gibbosus*, the Moroccan white seabream *Diplodus cadenati*, the striped seabream *Lithognathus mormyrus*, and the parrotfish *Sparisoma cretense*. The trend described by the GAM for the dusky grouper *Epinephelus marginatus* was slightly different than in the other four species, with a relatively stable decline in total length until around 1980, when a more pronounced size decrease began.

Table 2 presents, for each of the selected species, the slope *b*_1_′ of the trend line that best fits the various dispersions plotted on a logarithmic scale. Additionally, the rate of decline in total length over time was calculated for each species using the trend line (Table 2). As observed, longer-lived species such as the dusky grouper and the pink dentex have decline rates greater than 1 cm/year, due to their slower growth rates (Table 2).

**Table 2:** Slopes (*b*^*´*^_*1*_) and annual rates of decline in the average length of selected species.

Species	*b* ^*´*^_*1*_	Decrease in length (cm/year)
*Sparisoma cretense*	−0.017513	−0.52*
*Dentex gibbosus*	−0.022678	−1.19*
*Diplodus cadenati*	−0.01994	−0.43*
*Epinephelus marginatus*	−0.01933	−1.13*
*Lithognathus mormyrus*	−0.01364	−0.33*

*p-value<0.05.

The lengths at 50%maturity (L50), at 95% maturity (L95), and MLS were obtained from the literature (Table 3). The proposed MLS values for the Canary Islands [22–24] appear to be inconsistent with the lengths at maturity of these species. *Diplodus cadenati* is the only species for which MLS is greater than L50, thereby allowing 50% of the population to reach sexual maturity before being vulnerable to harvest. In the case of *Sparisoma cretense* and *Dentex gibbosus*, the MLS falls below the L50 for males, which poses a risk to the sustainability of these species. Additionally, *Epinephelus marginatus* shows first maturity lengths that are greater than its MLS; however the maturity sizes used for this species in the current regulations are based on studies from the Mediterranean because these studies are not available for Canary Islands. Finally, *Lithognathus mormyrus* currently has no established MLS. Curiously, mean catch sizes in the 2020s for all these species are below their respective L50 values (Fig 2).

**Table 3:** Sizes at first maturity (L50), mass maturity (L95), and minimum landing size (MLS, for Canary Islands) in centimeters for females and males of the selected species.

Species	*Females*		*Males*		*MLS*	*Reference*
	*L*_*50*_	*L*_*95*_	L_50_	L_95_		
*Sparisoma cretense*	21.3	33.2	23.3	31.7	22	[25]
*Dentex gibbosus*	34.7	47	38.6	55	35	[26]
*Diplodus cadenati*	21.6	26.2	20.1	25	22	[27]
*Epinephelus marginatus*	52-62		67-98		45	[28]
*Lithognathus mormyrus*	24.6	29	20.7	26	–	[29]

## 4. Discussion

The results obtained in this study show a clear decrease in the average size of fish over time—a pattern consistent with numerous previous works linking this phenomenon to sustained fishing pressure [16,17,30–33]. Recently some authors are associating this phenomenon with climate change, as an adaptive response to the increase in temperature [34,35], which would imply a very rapid evolutionary process of the species (sensus Hendry et al., 1999), in relatively few generations.

This phenomenon of decreasing fish size could be produced by management strategies, without to resort to multiple and complex microevolution phenomena in many species simultaneously. The comparison between legally established Minimum Landing Sizes (MLS) and lengths at 50% maturity (L50) reveals a concerning mismatch: in many cases, individuals can be caught and retained before reaching maturity. This practice not only jeopardizes the short-term sustainability of fish populations but could also promote artificial selection for individuals that mature at smaller sizes and younger ages [36,37].

The Canary Islands, as an isolated and small archipelago, can serve as a reference point for the evolution of fish populations. It has been observed that some commercially important demersal species are undergoing a significant reduction in the size of individuals being caught. Already in 2019, Jiménez-Alvarado et *al.* [17] highlighted this phenomenon in the waters around Gran Canaria, although their study primarily focused on the dusky grouper (*Epinephelus marginatus*). Our results not only confirm the trends identified by Jiménez-Alvarado et al. [17] but also show that this issue is more widespread than initially thought. The increase in the number of target species exploited by fishers may generate significant changes in affected marine ecosystems, potentially due to biomass reduction [38–40].

The data and trends presented in this work confirm the chronic overexploitation affecting many fish populations in the Canary Islands [40–43], particularly among species occupying higher trophic levels within benthic-demersal ecosystems. There is growing evidence that marine ecosystems in the Canary Islands are under severe pressure, facing chronic and intense overexploitation—especially over the last 50 years [17,40,41,44]. Historical photographs only serve to confirm this dramatic situation.

The size at 50% maturity (L50) has long been an arbitrary key reference point for setting minimum landing sizes in many fisheries. In theory, this could make sense: ensuring that at least half of the individuals in a population have the opportunity to reproduce before being caught should help support sustainability. However, in practice, this criterion falls short when we consider long-term effects, particularly evolutionary ones [45,46].

The main problem with L50 is that it leaves out the other 50% of the population—that is, all individuals that have not yet reached maturity. Legislation based solely on this threshold, without proper monitoring, could lead to undesirable evolutionary responses like the selection of genotypes that mature earlier and at smaller sizes while rarified those related with individuals that mature later and reach longer sizes, the larger spawners [47]. This type of fishery-induced evolution not only alters population structure but can also reduce future productivity and make populations more vulnerable to other factors such as climate change and environmental variability [48], even erasing part of the collective memory, that have the older specimens, and affecting the migratory patterns toward the best and furthest spawning areas [49].

In light of this, using the size at which 95% of individuals have reached maturity (L95) as a new reference for minimum landing sizes emerges as a necessary strategy. While this may seem like a huge change, its implications are substantial. By setting L95 as the limit, all individuals would have the chance to reproduce. This approach reduces pressure on slower-growing individuals and helps preserve greater genetic diversity within the population. Even so, this proposed improvement and the results of this study should be combined with a Harvest-slot length limits (based on species-specific ecological and demographic studies) to ensure the survival of megaspawners. Previous studies have also shown the need to increase MLS to protect more size classes of mature fish that can generate a larger source of viable larvae [50], and that these measures to increase MLS improve the conservation status of fish species against overfishing [51].

Knowing that adopting larger size limits such as L95, or higher, might result in smaller catches in the short term [52,53], which can be difficult to accept from an economic standpoint. But such decisions could mean the difference between a fishery collapsing within a few decades and one remaining productive and resilient over time [54]. “Incorporating evolutionary considerations into fisheries management is necessary for long term sustainability [28]. Critically, size-selective fisheries pressures can have irreversible evolutionary effects [55], reducing populations’ recovery potential even if fishing pressure is later reduced [56].”

In this context, our findings highlight the urgent need to revise current management criteria—particularly the re-evaluation of minimum landing sizes—to align them with the actual biological parameters of exploited species. Normally, fisheries regulations have set minimum sizes based on the L50, but as outlined above, this threshold may be insufficient to ensure long-term sustainability. To mitigate the risk of genetic overfishing, we propose not only establishing and determining the reproductive parameters of all exploited species for proper management but also expanding the convention of using L50 as MLS toward adopting L95. That is, ensuring that 95% of the population has reproduced at least once before being captured. This strategy not only secures greater reproductive contribution from the population before exploitation but also promotes the preservation of genetic variability and adaptive traits in target species.

## Supporting information

S1 Fig Examples of reference systems for measuring fish in photographs. The images show fish catches from different years, which can be measured using reference objects. In the first image, the reference point is the size of the floor tiles, which have remained the same in the city of Las Palmas for more than 50 years. In the second image, the reference point is the handle of the bucket, which has a standard size for all paint buckets.
(DOCX)

S2 Table Year, scientific name and estimated Total Length (cm) of the analysed individuals for the Canary Islands. This table includes all species analysed in the study.
(XLSX)
