# Supplementary figures and images for: Unintended evolutionary consequences of a minimum landing size regulations: Evidence and implications of fisheries-induced evolution
Source: PLoS One. 2026 Mar 2;21(3):e0343706. doi: 10.1371/journal.pone.0343706 (PMC12952581; doi:10.1371/journal.pone.0343706)

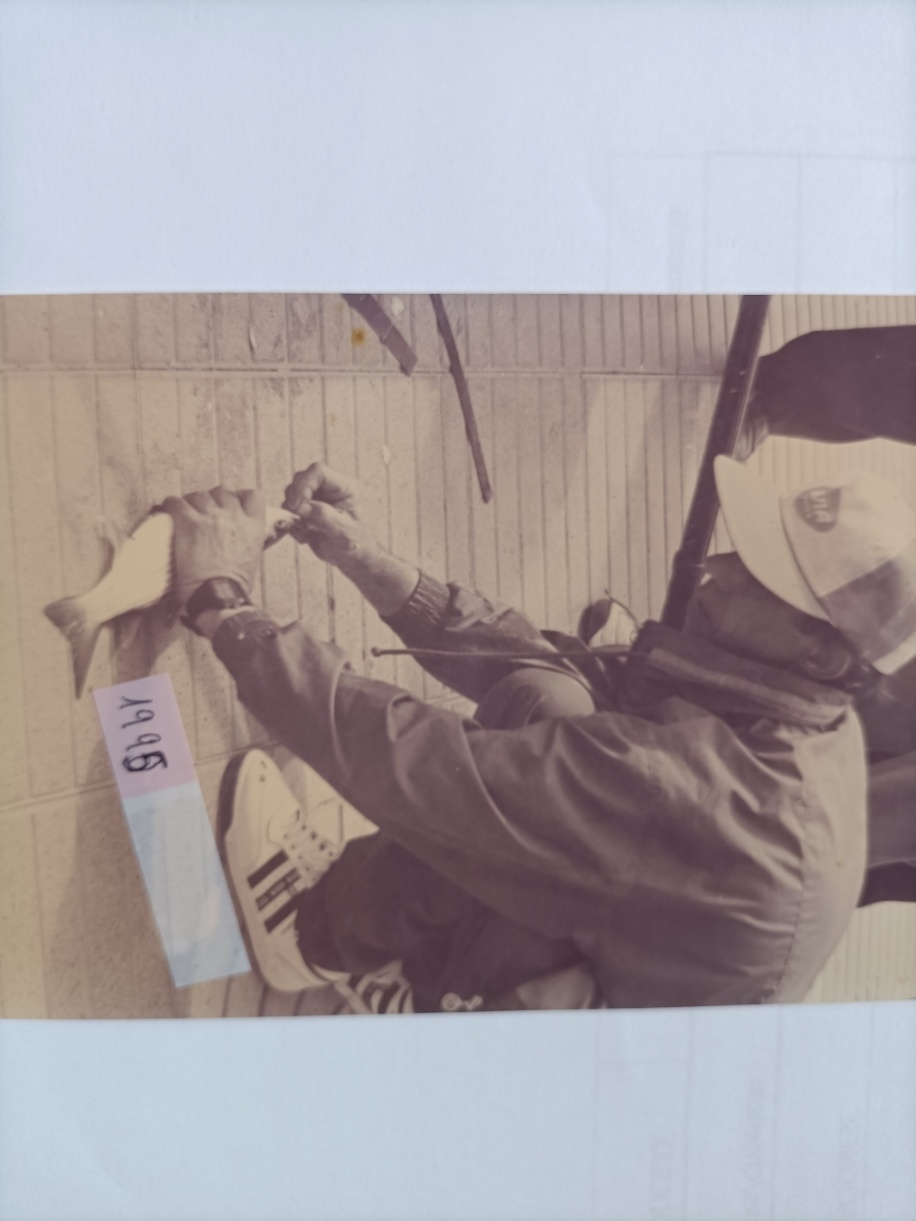

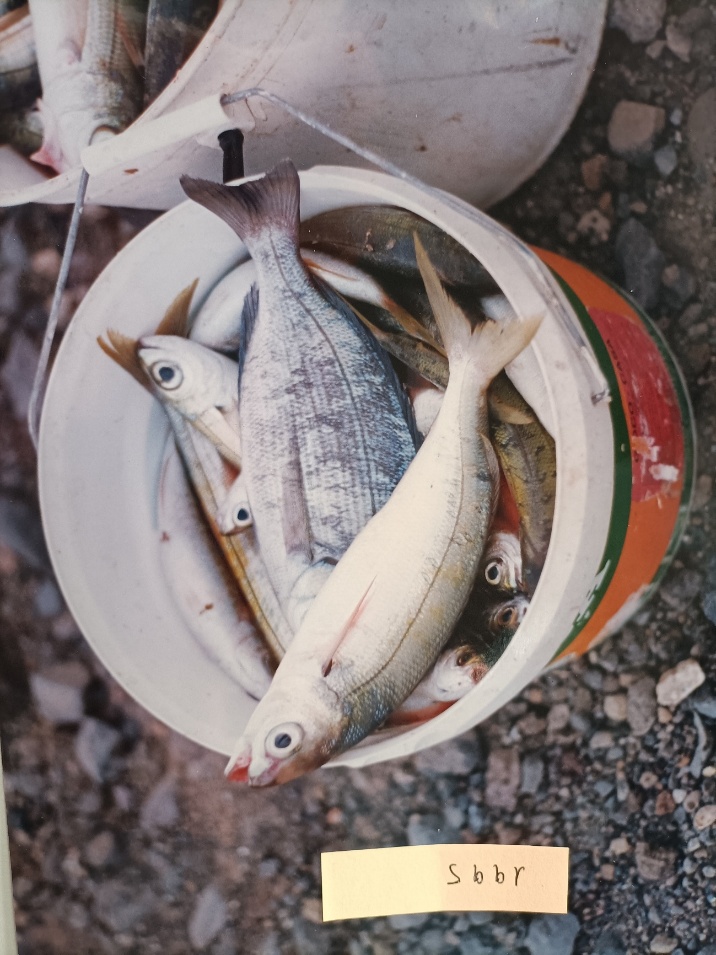

Supplement: S1 Fig — The images show fish catches from different years, which can be measured using reference objects. In the first image, the reference point is the size of the floor tiles, which have remained the same in the city of Las Palmas for more than 50 years. In the second image, the reference point is the handle of the bucket, which has a standard size for all paint buckets. (DOCX) [file pone.0343706.s001.docx]
